# Pharmacogenomics-assisted treatment versus standard of care in schizophrenia: a systematic review and meta-analysis

**DOI:** 10.1186/s12888-024-06104-4

**Published:** 2024-10-08

**Authors:** Saibal Das, Manoj Kalita, Manabendra Makhal, M Devaraja, Bhavani Shankara Bagepally, Jerin Jose Cherian, Rajesh Aadityan, Mounamukhar Bhattacharjee, Sarnendu Mondal, Sreyashi Sen, Manaswini Mondal, Aniruddha Basu, Atanu Kumar Dutta, Indranil Saha, Asim Saha, Amit Chakrabarti

**Affiliations:** 1 Indian Council of Medical Research - Centre for Ageing and Mental Health, Kolkata, India; 2 Department of Global Public Health, Karolinska Institutet, Stockholm, Sweden; 3 Department of Psychiatry, Calcutta National Medical College and Hospital, Kolkata, India; 4 Indian Council of Medical Research, National Institute of Epidemiology, Chennai, India; 5 Indian Council of Medical Research, New Delhi, India; 6 All India Institute of Medical Sciences, Raebareli, India; 7 Department of Psychiatry, All India Institute of Medical Sciences, Kalyani, India; 8 Department of Biochemistry, All India Institute of Medical Sciences, Kalyani, India

**Keywords:** Antipsychotic, Pharmacogenomics (PGx), Precision medicine, Schizophrenia, Therapeutic drug monitoring

## Abstract

**Background:**

Pharmacogenomic (PGx) factors significantly influence how patients respond to antipsychotic medications This systematic review was performed to synthesize the clinical utility of PGx-assisted treatment versus standard of care in schizophrenia.

**Methods:**

PubMed, Embase, and Cochrane CENTRAL databases were searched for randomized controlled trials (RCTs) from inception till June 2024 that had compared the clinical utility of PGx-assisted intervention as compared to the standard of care in schizophrenia. The primary outcome was safety, and the secondary outcomes were efficacy and medication adherence. Pooled standardized mean differences (SMD) along with a 95% confidence interval (CI) were calculated (random-effects model) wherever feasible.

**Results:**

A total of 18,821 studies were screened, and five were included for review. All the RCTs had a high risk of bias. Four studies included the commonly used antipsychotics. Three studies reported negative outcomes (safety, efficacy, and medication adherence) and two reported positive outcomes (safety) using different scales. In the meta-analysis, there were significant differences in the total Udvalg for Kliniske Undersogelser Side-Effect Rating scale score [SMD 0.95 (95% CI: 0.76–1.13), *p* < 0.001); I^2^ = 0%] and the total Positive and Negative Syndrome Scale score [SMD 10.65 (95% CI: 2.37–18.93), *p* = 0.01); I^2^ = 100%] between the PGx-assisted treatment and standard of care arms. However, the results were inconsistent, and the certainty of evidence (GRADE criteria) was very low.

**Conclusion:**

Current evidence on the clinical utility of PGx-assisted treatment in schizophrenia is limited and inconsistent and further evidence is required in this regard.

**Supplementary Information:**

The online version contains supplementary material available at 10.1186/s12888-024-06104-4.

## Background

The global burden of schizophrenia is 23.6 [95% confidence interval (CI): 20.2–27.2] million [1]. The effectiveness of antipsychotic treatment in individuals with schizophrenia varies greatly, making it impossible to foresee which patients will respond positively or negatively to the medication. Furthermore, around 20–50% of these patients do not respond well to antipsychotics leading to adverse repercussions for the patients, their families, and the broader society [2, 3]. Further, many antipsychotic medications have substantial adverse effects profiles [4]. The trial-and-error approach is frequently necessary prior to finding effective treatment, leading to treatment delays that can potentially affect patient adherence and worsen the disease [5].

Pharmacogenomic (PGx) factors significantly influence how patients respond to antipsychotic medications [6, 7]. It has been suggested that the selection of antipsychotics and adjustment of doses according to the genetic profile (presence of functional polymorphisms) of patients has the potential to significantly improve the safety profile and efficacy of antipsychotics [8]. Strong evidence supports the influence of cytochrome (CYP) variants on the development of drug-induced adverse reactions [9, 10]. Some randomized controlled trials (RCTs) have explored PGx-assisted intervention encompassing genotype-specific antipsychotic selection and/or dosing to aim for an optimal response; however, there is no summarized evidence in this regard. As this field is evolving, it is important to synthesize the available evidence and identify the evidence gaps to pave the way for future research. Hence, this systematic review was performed to evaluate the clinical utility of PGx-assisted treatment versus standard of care in schizophrenia.

## Methods

### Search strategy

A literature search was conducted in the MEDLINE/PubMed, Embase, and Cochrane CENTRAL electronic databases for interventional studies published in English from inception till June 2024. Various search terms were utilized, as outlined in Table S1. These search terms were adapted for different bibliographic databases, incorporating database-specific filters. Four independent authors identified relevant studies based on their titles and abstracts using the search strategy. The Rayyan software was used for this purpose. They then obtained the abstracts and, if necessary, the full texts of the articles to evaluate their suitability for inclusion. The discrepancies were resolved by discussion with another author who acted as an arbiter.

### Study selection

The authors included RCTs that had compared the clinical utility (safety and/or efficacy) of PGx-assisted intervention as compared to the standard of care in patients with schizophrenia. Those RCTs that had not included PGx information as a part of the initial intervention but performed genotyping post-hoc in a subgroup of patients guided by safety and/or efficacy response, those RCTs that had randomized patients based on their clinical response and genotyping results from an initial run-in period, and all kinds of observational studies evaluating genetic association with antipsychotic safety and/or efficacy were excluded. The primary outcome was safety, and the secondary outcomes were efficacy and medication adherence.

### Quality assessment

For the risk of bias analysis, the Cochrane risk of bias tool 2 [11] was used by an independent author. The tool includes the assessment of the following biases: randomization process, deviations from intended interventions, missing outcome data, measurement of the outcome, selection of the reported result, and overall.

### Data collection and analysis

A standardized, pre-formatted form was used to extract data from the eligible studies. The extracted information encompassed various aspects, including the details of interventions, comparators, and outcomes. The WebPlotDigitizer tool was used to extract information from the figures. Summary estimates were employed in the analysis, and meta-analysis was performed when sufficient and homogenous data were available for the outcomes. The statistical analysis was executed using RevMan version 5.3 software. Standardized mean differences with a corresponding 95% CI were determined for the differences in differences of the total Udvalg for Kliniske Undersogelser Side-Effect Rating scale (UKU-SERS) and Positive and Negative Syndrome Scale (PANSS) scores between the two groups using a random-effects model. If data were not sufficient to conduct a meta-analysis, qualitative/narrative summaries of the study findings were mentioned. The analysis also took into account attrition rates (dropouts, loss to follow-up, and withdrawals). A critical appraisal of missing data issues and the methods used for imputation were performed. Heterogeneity was analyzed by the Cochrane Q (χ^2^) test conducted on n–1 degree of freedom with a 5% α error for statistical significance and the I^2^ test was calculated. The I^2^ values were categorized as low (25%), medium (50%), and high (75%) [12].

### Certainty assessment

The GRADE (Grading of Recommendations Assessment, Development, and Evaluation) approach was used to assess the certainty of the generated evidence [13].

### Study protocol

This meta-analysis complies with the Preferred Reporting Items for Systematic Reviews and Meta-Analyses (PRISMA) guidelines. The study protocol was registered apriori in the International Prospective Register of Systematic Reviews (PROSPERO ID: CRD42024558949).

## Results

**Fig. 1:**
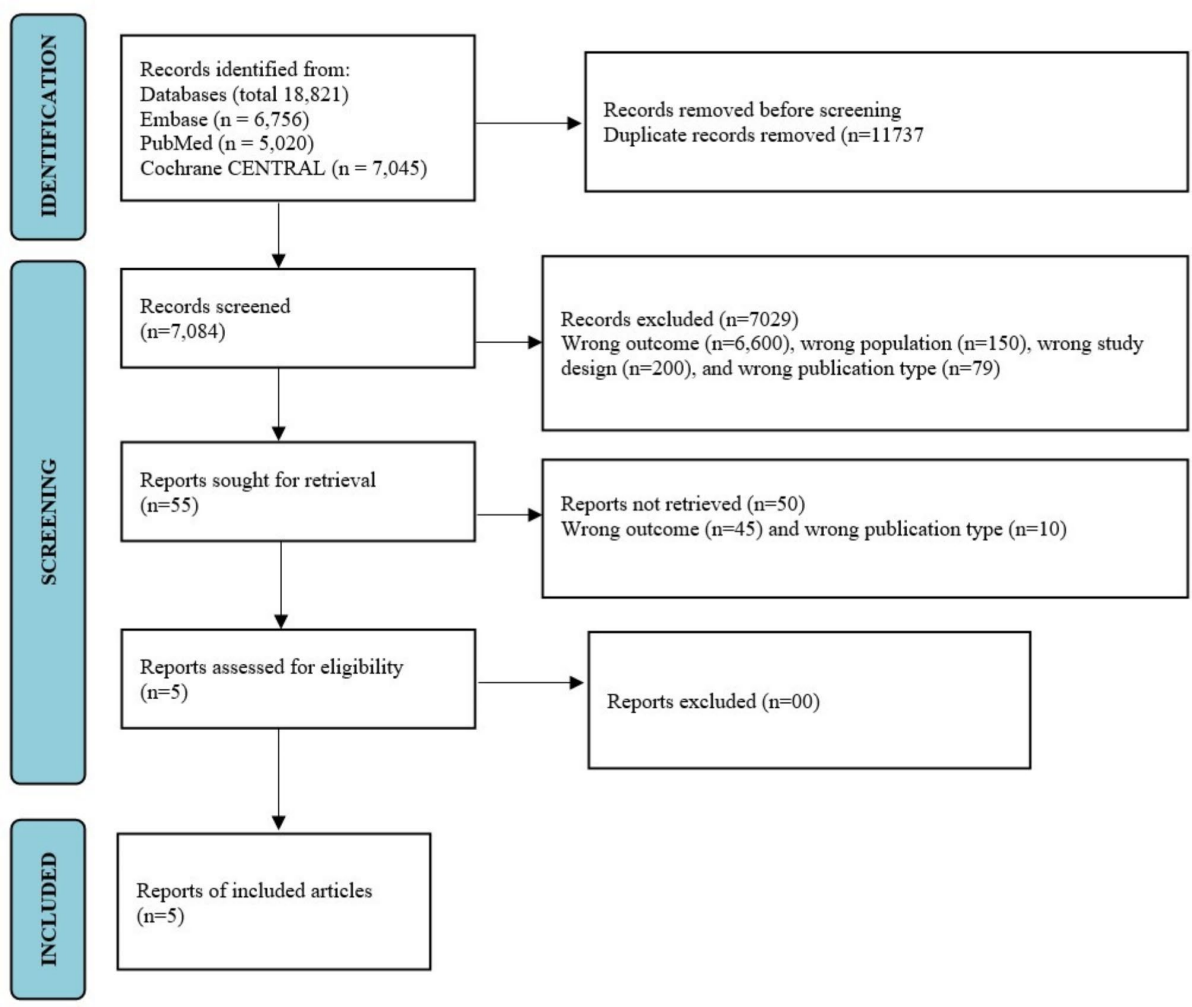
Study flowchart

A total of 18,821 articles were screened and, five articles [14–18] were included (Fig. 1) for systematic review. The results of the risk of bias analysis are itemized in Table S2. All the RCTs had a high risk of bias. The summary of the study characteristics is enumerated in Table 1. Four studies included the commonly used antipsychotics. CYP [14–16] and non-CYP [17, 18] polymorphism information was incorporated into the PGx-assisted intervention. The study duration ranged from three to twelve months. The scales used for safety were UKU-SERS and Common Terminology Criteria for Adverse Events (CTCAE). The scales used for efficacy were PANSS, Clinical Global Impressions (CGI), Scale for the Assessment of Positive Symptoms (SAPS), Global Assessment of Functioning (GAF), and Personal and Social Performance (PASP).

**Table 1:** Characteristics of the included studies

Author, year, country	*n* (intervention and comparator arms)	Patients	Intervention arm	Genotyping matrix and methods	Therapeutic drug monitoring	Comparator arm	Treatment duration	Primary outcome(s)	Secondary outcome(s)	Key findings
Arranz, et al., 2019, Spain [14]	123 and 167	Schizophrenia, schizoaffective, or delusional disorders (DSM-V)	Antipsychotic (clozapine, risperidone, olanzapine, paliperidone, aripiprazole, quetiapine, ziprasidone, trifluoperazine, haloperidol, asenapine, and pimozide) dosing according to respective *CYP1A2*, *CYP2D6*, *CYP2C19*, and *CYP3A5* polymorphisms (21variants)	• Blood • Commercial kit (iPlex^®^ Gold chemistry and MassARRAY platform), TaqMan probes, and technology for copy number variations	Performed for checking adherence	Standard of care	12 weeks	Adverse effects (UKU-SERS score)	Efficacy (PANSS score)	• Intervention did not lead to a significant reduction in the UKU-SERS score • There was no significant difference in PANSS score
Eadon, et al., 2023, USA [15]^*^	1,306 and 1,306	Psychosis	Antipsychotic (aripiprazole) dosing according to respective *CYP2D6* and *CYP3A4* polymorphisms (13 variants)	• Blood or saliva • PCR-based OpenArray^®^ (TaqMan™ Assay) and technology for copy number variations	Not performed	Standard of care	12 months	Adverse effects (National Institute of Health’s CTCAE)	Potential drug-drug interactions	• Significant reduction in the risk of serious adverse effects and death
Jurgens, et al., 2020, Denmark [16]	95, 94, and 101	Schizophrenic spectrum disorders (ICD-10)	Antipsychotic (clozapine, risperidone, olanzapine, aripiprazole, amisulpride, chlorprothixene, fluphenazine, flupentixol, quetiapine, penfluridol, ziprasidone, melperone, perphenazine, pimozide, haloperidol, levomepromazine, and zuclopenthixol) dosing according to *CYP2D6* and *CYP2C19* polymorphisms (7 variants)	• Blood • Tetra-primer and multiplex long PCR	Not performed	Structured clinical monitoring and standard of care	12 months	Antipsychotic drug adherence	Number of drug and dose changes, adverse effects (UKU-SERS score), and efficacy (SAPS score)	• Intervention did not lead to a significant difference in antipsychotic drug adherence and SAPS score • The effect on UKU-SERS score was inconsistent and the data was skewed • Sub-group analyses among extreme metabolizers showed similar results
Kang, et al., 2023, China [17]	113 and 97	Schizophrenia (DSM-IV) with a PANSS score of ≥ 60	Antipsychotic (amisulpride, aripiprazole, clozapine, olanzapine, paliperidone, quetiapine, risperidone, and ziprasidone) selection and dosing according to *CYP1A2*, *CYP2D6*, *CYP3A4*, *DRD2*, *EPM2A*, *HTR1A*, *HTR2A*, *HTR2C*, *MC4R*, *RGS4*, and *SH2B1* polymorphisms (26 variants)	• Saliva • MassArray (MALDI-TOF MS)	Not performed	Standard of care	12 weeks	Efficacy (PANSS score)	Response and symptomatic remission rates, chlorpromazine equivalent dose of antipsychotics	• Intervention demonstrated significantly higher changes in PANSS score, response rate, and remission rate • The intervention group required lower chlorpromazine equivalents of antipsychotics
Qin, et al., 2024, China [18]	109 and 77	Schizophrenia (ICD-11)	Antipsychotic (amisulpride, aripiprazole, clozapine, olanzapine, paliperidone, quetiapine, risperidone, and ziprasidone) dosing according to *CYP1A2*, *CYP2B6*, *CYP2C19*, *CYP2D6*,* CYP3A4*,* ABCB1*, *ADRA2A*, *ANKK1*, *COMT*, *DRD2*, *DRD3*, *EPM2A*, *FKBP5*, *GABRA1*, *GNB3*, *HTR1A*, *HTR2A*, *HTR2C*, *MC1R*, *MC4R*, *MDGA2*, *NAT2*, *RGS4*, *SACM1L*, *SH2B1*, *SLC6A4*,* UGT2B7* and *UGT2B15* polymorphisms (65 variants)	• Saliva • PCR, RFLP, and MALDI-TOF MS	Not performed	Standard of care	12 weeks	Efficacy (PANSS score and CGI score)	Social functioning (GAF score and PSP score)	• Intervention led to significant improvements in PANSS score, GAF score, PSP score, and relapse rate

^*^ The data related to antipsychotic (aripiprazole) are only included

CGI: Clinical Global Impressions, CPIC: Clinical Pharmacogenetics Implementation Consortium, CTCAE: Common Terminology Criteria for Adverse Events, DPWG: Dutch Pharmacogenetics Working Group, DSM: Diagnostic and Statistical Manual of Mental Disorders, GAF: Global Assessment of Functioning, ICD: International Classification of Diseases, MALDI-TOF MS: matrix-assisted laser desorption ionization time-of-flight mass spectrometry, PANSS: Positive and Negative Syndrome Scale, PCR: polymerase chain reaction, PSP: Personal and Social Performance, RFLP: restriction fragment length polymorphism, SAPS: Scale for the Assessment of Positive Symptoms, UKU-SERS: Udvalg for Kliniske Undersogelser Side-Effect Rating scale, USA: United States of America

**Fig. 2:**
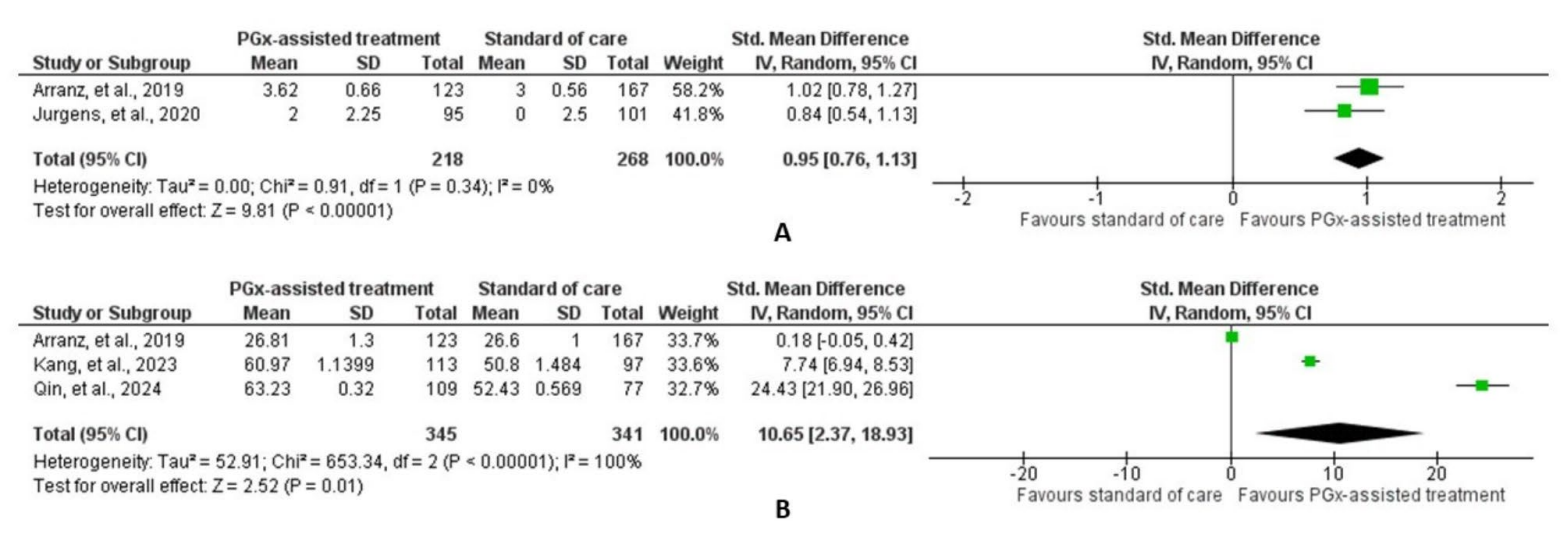
Forest plot showing the difference in the total Udvalg for Kliniske Undersogelser Side-Effect Rating scale score (**A**) and the Positive and Negative Syndrome Scale score (**B**) between the pharmacogenomics (PGx)-assisted treatment group and the standard of care group. For Jurgens, et al., 2020 study, the data of the PGx-assisted treatment and the standard of care groups were included. The median and interquartile range were approximated to mean and standard deviation

Arranz et al., 2019 [14] found that adjusting antipsychotic doses based on CYP polymorphisms did not significantly reduce adverse effects overall, but patients with certain *CYP2D6* and *CYP1A2*/*CYP2C19* variants showed notable improvements. Eadon et al., 2023 [15] reported no significant overall reduction in adverse effects or mortality with PGx-assisted interventions, though a significant reduction was observed for patients tested for aripiprazole. Jurgens et al., 2020 [16] found no significant benefits in adherence, safety, or efficacy with PGx-assisted treatment, while Kang et al., 2023 [17] and Qin et al., 2024 [18] showed significant improvements in efficacy and response rates in the Chinese population. In the meta-analysis, there was a significant difference in the UKU-SERS score [standardized mean difference, 0.95 (95% CI: 0.76–1.13), *p* < 0.001); I^2^ = 0%] and the PANSS score [standardized mean difference, 10.65 (95% CI: 2.37–18.93), *p* = 0.01); I^2^ = 100%] between the PGx-assisted treatment and standard of care arms at the end of the treatment period. (Fig. 2). Except for *CYP1A2* or *CYP2C19* extreme metabolizers and clozapine in the study by Arranz, et al., 2019 [14], no conclusive results could be obtained on other gene-drug pairs and extreme metabolizers in different populations. The certainty of evidence generated (GRADE criteria) was; however, very low for both safety and efficacy outcomes (Table S3–S5).

Arranz, et al., 2019 [14] compared the effects of adjusting antipsychotic doses based on CYP polymorphisms with the standard of care in the Spanish population. Although no significant differences in adverse effects were observed overall, the intervention group resulted in a greater reduction in adverse effects, though not statistically significant. Notably, patients in the intervention group carrying *CYP2D6* ultra-rapid or poor metabolizer variants and treated with *CYP2D6* substrates showed significantly higher improvements in various adverse effects compared to those in the standard-of-care group. Similarly, *CYP1A2* or *CYP2C19* ultra-rapid or poor metabolizer variant patients in the intervention group treated with clozapine showed a higher reduction in adverse effects although the differences were not statistically significant. The risk of bias in this study was high because the antipsychotic treatments and severity of symptoms were not evenly distributed in the groups post-randomization.

Eadon, et al., 2023 [15] conducted a pragmatic RCT in the United States of America using propensity matching and evaluated the clinical utility of PGx-assisted intervention for twenty-six drugs in different disease areas based on the guidelines of the Clinical Pharmacogenetics Implementation Consortium or the Dutch Pharmacogenetics Working Group. For patients with psychotic disorders, aripiprazole and its related genes were included. Overall, the intervention did not significantly reduce the occurrence of adverse effects or mortality. However, subgroup analyses indicated a significant reduction in serious adverse effects and mortality for those tested for aripiprazole. The risk of bias in this study was high because of the pragmatic nature.

In the study by Jurgens, et al., 2020 [16], Danish patients were randomly allocated to PGx-assisted intervention, structured clinical monitoring, or standard of care groups using a predictive enrichment design. No significant improvements in antipsychotic drug adherence, safety, or efficacy were observed by PGx-assisted treatment as compared to the other groups. Kang, et al., 2023 [17] and Qin, et al., 2024 [18] compared the effects of selecting antipsychotics and adjusting doses based on CYP and non-CYP polymorphisms with standard of care in the Chinese population. The intervention led to a significant improvement in efficacy (schizophrenia symptoms and social functioning), response rate, and remission rate as compared to the standard of care, and the effect was noticed before the trial was completed. The studies by Jurgens, et al., 2020 [16], Kang, et al., 2023 [17], and Qin, et al., 2024 [18] were single-blind studies, physicians were not masked in the study groups, which might increase the clinicians’ attention toward the patient’s medical treatment. Also, in the study by Qin, et al., 2024 [18] more patients who had reached remission or response levels of efficacy dropped out or were lost to follow-up.

## Discussion

In this systematic review, it was found that, overall, there were mixed results in terms of safety and efficacy between PGx-assisted treatment and standard of care in patients with schizophrenia. The studies by Arranz, et al., 2019 [14] and Jurgens, et al., 2020 [16] showed negative results. Although Eadon, et al., 2023 [15] showed positive results, only aripiprazole and a limited number of genes and polymorphisms were included, and thus, a comprehensive PGx-assisted treatment algorithm was not used. Two RCTs conducted in the Chinese population showed significant improvement in the efficacy outcomes following PGx-assisted treatment as compared to the standard of care [17, 18]. These studies used different formulas to estimate the efficacy outcomes (PANSS score) and safety outcomes were not reported in these studies. None of the studies utilized therapeutic drug monitoring to ensure clinical response. Also, the risk of bias in three of the included RCTs was high. In our meta-analysis, it was found that PGx treatment led to a significant improvement in the UKU-SERS and PANSS scores as compared to the standard of care. However, not all studies could be included in the meta-analysis and because of the inconsistencies, the GRADE of evidence generated was very low.

Optimizing medication, such as using PGx-assisted treatment, for individuals with psychotic disorders who are on multiple medications is complex and limited. Therefore, a more holistic and integrated approach is necessary [19]. Optimizing treatment through precision medicine is essential to enhance efficacy and minimize adverse drug reactions in patients undergoing antipsychotic therapy. A key objective of precision medicine in this context is to utilize genetic information to improve safety, efficacy, and outcomes. Pharmacogenomic (PGx) testing serves as a companion decision-support tool, considering all relevant individual clinical and demographic data. Presently, the United States Food and Drug Administration includes information on PGx biomarkers in the labeling of nine antipsychotic drugs [20]. Similarly, the Pharmacogenomics Knowledge Base website identifies ten antipsychotic drugs that should be used with caution in patients who are poor CYP2D6 metabolizers [21]. Drug labels for certain antipsychotics include pharmacogenomic (PGx) information. The Royal Dutch Association for the Advancement of Pharmacy-Dutch Pharmacogenetics Working Group has issued PGx dosing guidelines for six antipsychotics based on CYP2D6 genotypes [22, 23]. Guidelines from the Clinical Pharmacogenetics Implementation Consortium [24] and the Canadian Pharmacogenomics Network for Drug Safety [25] also offer expert recommendations on drug dosing and selection for drug-gene pairs. These guidelines are based on strong evidence supporting the advantages of PGx testing with antipsychotics.

Functional candidate gene variants have been extensively studied in a variety of antipsychotic response phenotypes in the treatment of schizophrenia [26]. Multiple alleles of genes involved in pharmacokinetics (particularly isoenzymes of CYP450), as well as variants of genes involved in dopamine, serotonin, and glutamate neurotransmission, have already been identified as ones of significant impact on antipsychotic response in candidate-gene approach studies and genome-wide association studies [27]. Multiple observational studies have investigated genetic association with antipsychotic responses and/or adverse effects [6, 28]. As the size of genome-wide association study samples has increased, more genes have been identified with high confidence that has begun to provide insight into the etiological and pathophysiological foundations of this disorder [29, 30]. RCTs, employing alternative designs, such as performing post-hoc genotyping after the initial trial or randomizing patients following clinical assessment and genotyping in a run-in period based on their clinical response and genotyping results from an initial run-in period, were also performed previously [8, 31–33]. In this review, RCTs that included PGx information as a part of the intervention were included. The results were either in favor of PGx-assisted prescribing or showed no difference between PGx-assisted treatment and standard of care for clinical outcomes. Regarding cost-effectiveness, PGx might offer benefits over the standard of care in schizophrenia management [34, 35].

There are several commercially available pharmacogenetic tests that interrogate CYP functional polymorphisms, which can be potentially useful for the personalization of antipsychotic drugs [36]. However, despite growing evidence showing the influence of genetic factors on antipsychotic treatment efficacy, PGx information is rarely used in clinical settings for the personalization of treatment in psychotic disorders although several studies have shown that most clinicians and patients are in favor of PGx-assisted treatment [37, 38]. It is also important to identify the relevant polymorphisms from the pharmacokinetic and pharmacodynamic attributes of the respective antipsychotic medicines and include the information in the treatment (antipsychotic selection and dosing) algorithm. The most robust results are those that associate CYP functional polymorphisms with adverse reactions [39], whereas other polymorphisms in dynamic genes have been also proven to be useful for the improvement of antidepressant medication [40].

PGx-assisted treatment has a number of drawbacks as well. From a decision-making perspective, the CYP test is not a one-step solution that relates one test result to a therapeutic target dose [41]. The phenotypic distinction of individuals with different CYP genotypes may be blurred because of an overlap between pharmacokinetic parameters and possible phenotype conversion. Attempts to estimate dose adjustments for psychotropic drugs are based on average doses adjusted to mean kinetic parameters or to numbers of functional alleles [42]. PGx-assisted treatment allows for detecting more potential pharmacogenetic problems but also creates the possibility for more erroneous medical decisions and might, in theory, lead to unjustifiably withholding effective pharmacological treatment from the patient. There are also inconsistencies across guidelines for dose adjustment of *CYP2D6-* and *CYP2C19*-dependent drugs [43, 44].

When designing pharmacogenomic testing panels for diverse populations, it is crucial to consider variations in allele frequencies and tailor the design accordingly. There have been ongoing discoveries of new genes and loci associated with the efficacy and adverse effects of antipsychotic medications. These factors should be considered for inclusion in the design of PGx testing. In addition, the contribution of each gene-drug pair to the treatment outcome needs to be evaluated. For all these reasons, it is often difficult to obtain consent from patients before administering PGx-assisted treatment. On the other hand, routine implementation of therapeutic drug monitoring can be an alternative cheaper technique to predict variability in response to antipsychotics. Interestingly, none of the included studies [14–18] utilized therapeutic drug monitoring to ensure clinical response [45, 46]. There are specific evidence gaps in terms of the overall clinical utility (safety and efficacy) of PGx-assisted treatment in schizophrenia, especially with regards to specific gene-drug pairs and extreme metabolizers in varied populations, the cost-effectiveness of PGx-assisted treatment, and the facilitators and barriers of implementing of PGx-assisted treatment in routine clinical practice. Studies are underway, including one from our group to address some of these gaps [47–49].

One systematic review has been recently published on this topic; however, the authors have included both RCTs and observational studies [8]. The strengths of our study are the inclusion of only RCTs involving diverse populations from the literature and the use of a robust methodology for analysis. Several limitations were identified in the studies that were included. All the studies had a high risk of bias. The sample sizes were small, and hence, the proportion of patients having different genetic variants was even smaller to make a meaningful comparison. The chance of publication bias could not be eliminated. Also, all important antipsychotics and genotypes were not evaluated in all studies, and physicians’ interpretations of the PGx-assisted treatment algorithm based on genotyping results varied. Caution is warranted in generalizing the findings of the results of one population to the other due to variations in the genetic constitutions. Factors such as smoking and coffee consumption, which could affect the activity of the CYP enzymes, as well as concomitant nonpsychiatric medications were not accounted for due to limitations in the data. Furthermore, there were limitations in our review process. Data for all the desired outcomes were either unavailable, resulting in their exclusion from the review, or the inability to include them in the meta-analysis. This was mostly because of the variations in the outcome parameters and differences in the scales used. Only descriptive statistics were used for these data. Additionally, heterogeneity was observed in some outcomes, likely due to variations in the designing and implementation of treatment algorithms due to differences in genotyping and antipsychotics, treatment duration, and variations in the outcomes and corresponding scales used. This may lead to inconsistent findings and limit the generalizability of the results. Notwithstanding these limitations, to the best of our knowledge, this is the first systematic review and meta-analysis to synthesize the evidence on the clinical utility of PGx-assisted treatment as compared to the standard of care in schizophrenia.

## Conclusion

The clinical utility of PGx-assisted treatment as compared to the standard of care in schizophrenia in terms of safety and efficacy has been conflictingly reported in the literature. These are variations in the results based on the studied population, antipsychotic drugs studied, and genes and variations included in the treatment algorithm. Hence, current evidence on the clinical utility of PGx-assisted treatment in schizophrenia is limited and is of a very low GRADE. High-quality large studies are warranted in this regard. The clinical utility, cost-effectiveness, facilitators, and barriers of PGx-assisted treatment in schizophrenia need to be evaluated in the future in large RCTs in various populations.

## Electronic supplementary material

Below is the link to the electronic supplementary material.

Supplementary Material 1

Supplementary Material 2

## Data Availability

All data related to this article are included within the main and supplementary files.
