# Supplementary material for: Pharmacogenomics-assisted treatment versus standard of care in schizophrenia: a systematic review and meta-analysis
Source: BMC Psychiatry. 2024 Oct 8;24:663. doi: 10.1186/s12888-024-06104-4 (PMC11463053; doi:10.1186/s12888-024-06104-4)
Supplement: Supplementary file 1 — Supplementary Material 1 [file 12888_2024_6104_MOESM1_ESM.docx]

**Supplementary Materials**

**Table s1.** Search strings used during literature search across three databases.

| **PubMed** |
| --- |
| **(pharmacogenetics[Title/Abstract]) OR (pharmacogenomics[Title/Abstract]) OR (polymorphism[Title/Abstract]) OR (single nucleotide polymorphism[Title/Abstract]) OR (mutation[Title/Abstract]) OR (personalized therapy[Title/Abstract]) OR (Cytochrome[Title/Abstract]) or (Pharmacogenetic intervention[Title/Abstract]) OR (pharmacogenetic testing[Title/Abstract])** |
| **(Schizophrenia[Title/Abstract]) OR (Schizophrenic Disorders[Title/Abstract]) OR (Disorder, Schizophrenic[Title/Abstract]) OR (Dementia Praecox[Title/Abstract]) OR (Schizophrenia Spectrum[Title/Abstract]) OR (Schizophrenia, Paranoid[Title/Abstract]) OR (Paranoid Schizophrenias[Title/Abstract]) OR (Delusional Disorder[Title/Abstract]) OR (Delusional Disorders[Title/Abstract]) OR (Schizophrenia, Disorganized[Title/Abstract]) OR (Disorganized Schizophrenia[Title/Abstract]) OR (Schizophrenias, Disorganized[Title/Abstract]) OR (Hebephrenic Schizophrenia[Title/Abstract]) OR (Schizophrenia, Childhood[Title/Abstract]) OR (Schizophrenia, Catatonic[Title/Abstract]) OR (Schizotypal Personality Disorder[Title/Abstract]) OR (Disorders, Schizotypal Personality[Title/Abstract]) OR (Schizophrenia, Latent[Title/Abstract]) OR (Latent Schizophrenias[Title/Abstract]) OR (Pseudopsychopathic Schizophrenia[Title/Abstract]) OR (Pseudopsychopathic Schizophrenias[Title/Abstract]) OR (Schizophrenia, Borderline[Title/Abstract]) OR (Borderline Schizophrenias[Title/Abstract]) OR (Incipient Schizophrenia[Title/Abstract]) OR (Incipient Schizophrenias[Title/Abstract]) OR (Schizophrenia, Pseudoneurotic[Title/Abstract]) OR (Pseudoneurotic Schizophrenias[Title/Abstract]) OR (Psychotic Disorders[Title/Abstract]) OR (Disorder, Psychotic[Title/Abstract]) OR (Psychosis[Title/Abstract]) OR (Psychoses[Title/Abstract]) OR (Schizoaffective Disorder[Title/Abstract]) OR (Disorders, Schizoaffective[Title/Abstract]) OR (Schizophreniform Disorders[Title/Abstract]) OR (Disorder, Schizophreniform[Title/Abstract]) OR (Psychosis, Brief Reactive[Title/Abstract]) OR (Brief Reactive Psychoses[Title/Abstract])** |
| **Embase** |
| ('pharmacogenetics':ab,ti OR 'pharmacogenomics':ab,ti OR 'polymorphism':ab,ti OR 'single nucleotide polymorphism':ab,ti OR 'mutation':ab,ti OR 'personalized therapy':ab,ti OR 'cytochrome':ab,ti OR 'pharmacogenetic intervention':ab,ti OR 'pharmacogenetic testing':ab,ti) |
| 'schizophrenia':ab,ti OR 'schizophrenic disorders':ab,ti OR 'disorder, schizophrenic':ab,ti OR 'dementia praecox':ab,ti OR 'schizophrenia spectrum':ab,ti OR 'schizophrenia, paranoid':ab,ti OR 'paranoid schizophrenias':ab,ti OR 'delusional disorder':ab,ti OR 'delusional disorders':ab,ti OR 'schizophrenia, disorganized':ab,ti OR 'disorganized schizophrenia':ab,ti OR 'schizophrenias, disorganized':ab,ti OR 'hebephrenic schizophrenia':ab,ti OR 'schizophrenia, childhood':ab,ti OR 'schizophrenia, catatonic':ab,ti OR 'schizotypal personality disorder':ab,ti OR 'disorders, schizotypal personality':ab,ti OR 'schizophrenia, latent':ab,ti OR 'latent schizophrenias':ab,ti OR 'pseudopsychopathic schizophrenia':ab,ti OR 'pseudopsychopathic schizophrenias':ab,ti OR 'schizophrenia, borderline':ab,ti OR 'borderline schizophrenias':ab,ti OR 'incipient schizophrenia':ab,ti OR 'incipient schizophrenias':ab,ti OR 'schizophrenia, pseudoneurotic':ab,ti OR 'pseudoneurotic schizophrenias':ab,ti OR 'psychotic disorders':ab,ti OR 'disorder, psychotic':ab,ti OR 'psychosis':ab,ti OR 'psychoses':ab,ti OR 'schizoaffective disorder':ab,ti OR 'disorders, schizoaffective':ab,ti OR 'schizophreniform disorders':ab,ti OR 'disorder, schizophreniform':ab,ti OR 'psychosis, brief reactive':ab,ti OR 'brief reactive psychoses':ab,ti |
| **COCHRANE** |
| **(Schizophrenia) OR (Schizophrenic Disorders) OR (Disorder, Schizophrenic) OR (Dementia Praecox) OR (Schizophrenia Spectrum) OR (Schizophrenia, Paranoid) OR (Paranoid Schizophrenias) OR (Delusional Disorder) OR (Delusional Disorders) OR (Schizophrenia, Disorganized) OR (Disorganized Schizophrenia) OR (Schizophrenias, Disorganized) OR (Hebephrenic Schizophrenia) OR (Schizophrenia, Childhood) OR (Schizophrenia, Catatonic) OR (Schizotypal Personality Disorder) OR (Disorders, Schizotypal Personality) OR (Schizophrenia, Latent) OR (Latent Schizophrenias) OR (Pseudopsychopathic Schizophrenia) OR (Pseudopsychopathic Schizophrenias) OR (Schizophrenia, Borderline) OR (Borderline Schizophrenias) OR (Incipient Schizophrenia) OR (Incipient Schizophrenias) OR (Schizophrenia, Pseudoneurotic) OR (Pseudoneurotic Schizophrenias) OR (Psychotic Disorders) OR (Disorder, Psychotic) OR (Psychosis) OR (Psychoses) OR (Schizoaffective Disorder) OR (Disorders, Schizoaffective) OR (Schizophreniform Disorders) OR (Disorder, Schizophreniform) OR (Psychosis, Brief Reactive) OR (Brief Reactive Psychoses)** |
| **(pharmacogenetics) OR (pharmacogenomics) OR (polymorphism) OR (single nucleotide polymorphism) OR (mutation) OR (personalized therapy) OR (Cytochrome) or (Pharmacogenetic intervention) OR (pharmacogenetic testing)** |

**Table s2.** Results of the risk of bias assessment using the Cochrane risk of bias tool 2.

| **Author, year** | **Bias arising from the randomization process** | **Bias due to deviations from intended interventions** | **Bias due to missing outcome data** | **Bias due to measurement of the outcome** | **Bias in selection of the reported result** | **Overall bias** |
| --- | --- | --- | --- | --- | --- | --- |
| Arranz, et al., 2019 | High | Low | Low | Low | Low | High |
| Eadon, et al., 2023 | Low | Low | High | High | High | High |
| Jurgens, et al., 2020 | Low | Low | Low | High | Low | High |
| Kang, et al., 2023 | Low | Low | Low | High | Low | High |
| Qin, et al., 2024 | Low | Low | Low | High | High | High |

**Table s3.** Certainty of the evidence for the safety and efficacy outcomes as per the GRADE (Grading of Recommendations Assessment, Development and Evaluation) approach.

| **GRADE domain** | **Judgement** | **Concerns about certainty domains** |
| --- | --- | --- |
| Methodological limitations of the studies | All studies had a serious risk of bias. | Serious |
| Indirectness | The population, intervention, and comparator in all studies provide direct evidence to the clinical question at hand. The intervention varied due to the variations in designing and implementation of treatment algorithm due to differences in genotyping and antipsychotics. The outcome assessment was also measured using different scales across the studies. | Serious |
| Imprecision | Some studies reported improvements, while some did not. The sample size varied widely. | Serious |
| Inconsistency | The direction and magnitude of the effect of intervention varied across different studies. The scales used were also different for each studies. | Serious |
| Publication bias | This was not suspected because both negative and positive studies were published, and the search strategy was robust. | Not suspected |

**Table s4.** Summary of findings for UKU-SERS and PANSS score outcomes according to the GRADE (Grading of Recommendations Assessment, Development and Evaluation) approach.

|  | | | | | | | | | | | |
| --- | --- | --- | --- | --- | --- | --- | --- | --- | --- | --- | --- |
| **Certainty assessment** | | | | | | | **Summary of findings** | | | | |
| **Participants (studies) Follow-up** | **Risk of bias** | **Inconsistency** | **Indirectness** | **Imprecision** | **Publication bias** | **Overall certainty of evidence** | **Study event rates (%)** | | **Relative effect (95% CI)** | **Anticipated absolute effects** | |
|  |  |  |  |  |  |  | **With [comparison]** | **With [intervention]** |  | **Risk with [comparison]** | **Risk difference with [intervention]** |
| **UKU-SERS score** | | | | | | | | | | | |
| 486 (2 RCTs) | serious^a^ | not serious | not serious | very serious | none | ⨁◯◯◯ Very low | 268 | 218 | - | - | SMD **0.95 SD higher** (0.76 higher to 1.13 higher) |
| **PANSS score** | | | | | | | | | | | |
| 686 (3 RCTs) | serious^a^ | serious^b^ | not serious | very serious^b^ | none | ⨁◯◯◯ Very low | 341 | 345 | - | - | SMD **10.65 SD higher** (2.37 higher to 18.93 higher) |

**CI:** confidence interval; **SMD:** standardised mean difference

#### Explanations

a. Antipsychotic treatments and severity of symptoms were not evenly distributed in the groups post-randomization, single-blind studies leading to bias in outcome assessment

b. Different studies showed different effect sizes

**Table s5.** Summary of findings for all outcomes according to the GRADE (Grading of Recommendations Assessment, Development and Evaluation) approach.

| **Outcome** | **Effect** | **Number of studies** | **Certainty in the evidence^*^** |
| --- | --- | --- | --- |
| Safety | There were heterogeneities in the population, intervention, and outcome assessment. The studies showed inconsistent effect | 3 | VERY LOW ⊕OOO (due to serious risk of methodological limitations, imprecision, and inconsistency) |
| Efficacy | There were heterogeneities in the population, intervention, and outcome assessment. The studies showed inconsistent effect | 4 | VERY LOW ⊕OOO (due to serious risk of methodological limitations, imprecision, and inconsistency) |
